# Effects of Storage Conditions on Consumer and Chemical Assessments of Raw ‘Nonpareil’ Almonds Over a Two‐Year Period

**DOI:** 10.1111/1750-3841.14055

**Published:** 2018-01-22

**Authors:** Emily A. Pleasance, William L. Kerr, Ronald B. Pegg, Ruthann B. Swanson, Anna N. Cheely, Guangwei Huang, Daniel R. Parrish, Adrian L. Kerrihard

**Affiliations:** ^1^ Dept. of Foods and Nutrition, College of Family and Consumer Sciences The Univ. of Georgia 305 Sanford Drive Athens GA 30602 U.S.A; ^2^ Dept. of Food Science & Technology, College of Agricultural and Environmental Sciences The Univ. of Georgia 100 Cedar St. Athens GA 30602 U.S.A; ^3^ Almond Board of California 1150 Ninth St., Suite, 1500 Modesto CA 95354 U.S.A; ^4^ Dept. of Nutrition and Food Studies, College of Education and Human Services Montclair State Univ. 1 Normal Ave. Montclair NJ 07043 U.S.A

**Keywords:** almonds, consumer acceptability, oxidation, sensory analysis

## Abstract

**Abstract:**

Raw almonds are a major commodity, yet much is unknown about how storage conditions determine their shelf life. The storage stability, as measured by consumer assessments and chemical measures, of raw almonds was determined for samples stored in cardboard boxes and polypropylene packaging for 2 years at 4, 15, 25, and 35 °C, and at 50% and 65% relative humidity (RH). Samples stored in unlined cartons always failed (>25% rejection) before their counterparts stored in polypropylene bags under identical environmental conditions. Models determined that polypropylene packaging (as opposed to unlined cardboard cartons) extended the time until sample rejection by more than 7 months. Temperature and RH were both negatively associated with storage time until failure. Flavor was a greater contributor to consumer acceptability than texture or odor, while peroxide values and free fatty acids were of greater importance in predicting raw almond consumer quality than measures of conjugated dienes or 2‐thiobarbituric acid‐reactive substances.

**Practical Application:**

The results of this study will allow almond producers to determine packaging types and environmental storage conditions that provide shelf life of a specified time.

## Introduction

Increased consumption of tree nuts has been linked to numerous health benefits, including reduced risk of cardiovascular disease, type‐2 diabetes, and obesity (Albert, Gaziano, Willett, & Manson, 2002; Berryman, West, Fleming, Bordi, & Kris‐Etherton, 2015; Jaceldo‐Siegl, Sabaté, Rajaram, & Fraser, 2004; Martínez‐González & Bes‐Rastrollo, 2010). Among tree nuts, almonds are the most abundantly produced in the world (International Nut and Dried Fruit Foundation, 2010). In the United States, the consumption of almonds is over twice that of walnuts, hazelnuts, pecans, and pistachios combined (Almond Board of California, 2016). Furthermore, almond sales have increased over the last few decades, as the *per capita* consumption more than quadrupled between 1980 and 2014 (USDA, 2014).

The high content of unsaturated fatty acids found in almonds, like most nuts, makes them susceptible to oxidation and to important quality losses if stored improperly or for too long. However, the specific rate of oxidation and flavor deterioration of a nut will be dependent on the distribution of individual fatty acids and other factors (Hudson & Gordon, 1999; Shahidi & John, 2013). In general, almonds maintain quality throughout storage better than some nuts, due to their low‐moisture and high antioxidant content (Huang, 2014; Shahidi & John, 2013).

Low‐moisture almonds are generally resistant to microbiological spoilage; thus, their shelf life is primarily defined by changes in sensory attributes (Hough, Langohr, Gómez, & Curia, 2003). The maintenance of appropriate crispness and chewiness levels is vital to acceptable texture characteristics. In conjunction, the development of rancidity and odors related to oxidation are detrimental to almond quality. Furthermore, because of their high lipid content, nuts may absorb odors during storage, resulting in them becoming less acceptable to consumers (Kader, 2013). It is well known that the overall development of off‐flavors and off‐odors, as well as the deterioration of texture, contributes significantly to degradation of the sensorial quality of many foods until it reaches a critical point at which the product becomes unacceptable to the consumer (Velasc, Dobarganes, & Márquez‐Ruiz, 2010).

As harvest season for almonds occurs only once per year, the determination of optimal storage conditions is important to the prevention of deterioration in quality during storage and shipment (Shahidi & John, 2013). Over 82% of the world's almond production occurs in California, with approximately 2/3 of the almonds being shipped internationally (Almond Board of California, 2015). It means that maintenance of quality throughout long‐distance transportation is of great importance. Harvested almonds are also transported and marketed in a variety of forms, which influence product stability. These forms include in‐shell, shelled kernels, and peeled seeds; whole or nut pieces; and raw and roasted nuts (Harris & Ferguson, 2013; Shahidi & John, 2013). It is known that the degree of processing plays a role in the rate of quality deterioration due to oxidation or rancidity development (Huang, 2014).

Almonds are typically held in bins, silos, or other bulk containers. The Almond Board of California recommends almonds be stored under cool, dry conditions (<10 °C/50 °F and <65% relative humidity [RH]), in which case the whole natural almonds can be stored for 2 years without a significant decrease in quality (Almond Board of California, 2016; Huang, 2014). It is also recommended that almonds be protected from oxygen, either through nitrogen flushing and/or vacuum packaging and that the nuts should avoid exposure to strong odors that might be absorbed (Almond Board of California, 2015).

There is little data, however, on how almonds stored in different packaging and under various environmental conditions are perceived and accepted by consumers. The objectives of this research were to determine if storage at different temperatures and humidities, or in packages of different moisture and oxygen permeabilities, would impact the quality of raw almonds throughout extended storage. Acceptability was determined by consumer assessments of almonds stored in varying storage conditions throughout a 24‐month period. In addition, chemical measures of oxidation and moisture transfer were determined along with physical measures of texture changes. Additional objectives of this research were to model the relationship between specific consumer assessment measures and overall acceptability for stored raw almonds, and to model the relationship between chemical assessments and consumer assessment outputs.

## Materials and Methods

### Study design

The study consisted of an incomplete factorial design in which almonds were stored under varying conditions over a 24‐month period and tested by chemical means and by consumer sensory evaluation. Almonds were stored in either unlined cardboard cartons (UC) or in sealed polypropylene bags (PPB). Storage temperatures were 15, 25, and 35 °C at RH levels of 50% or 65%. Samples were also held at 4 °C without RH control. This resulted in 13 different conditions as outlined in Table 1.

**Table 1:** Parameters for storage of raw almond samples (13 total storage conditions)

Packaging	Temperature (°C)	Relative humidity (%)
Sealed polypropylene bags (PPB)	4	No RH control
	15	50
		65
	25	50
		65
	35	50
		65
Unlined cardboard cartons (UC)	4	No RH control
	15	50
	25	50
		65
	35	50
		65

**Figure 1:**
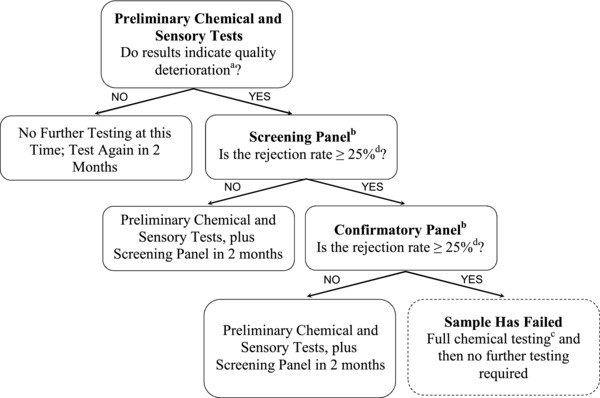
Process flow and decision making for chemical, instrumental, and sensory testing. ^a^PVs > 2.0 meq. active O_2_/kg oil or detection of off‐sensory notes by 3 experienced sensory analysts. ^b^Response of “No” to “If you had purchased this product would you eat it?” ^c^PV, FFA, CD, TBARS, *A_w_*, and MC.

Figure 1 depicts a flowchart for the various sample analyses, and how these were triggered at various times during the storage period. Prior to packaging and storage, almonds were evaluated at baseline by a consumer sensory panel (*n* = 118) as well as by chemical means. The chemical tests included peroxide value (PV), free fatty acid (FFA), conjugated dienoic acid (CD), 2‐thiobarbituric acid‐reactive substances (TBARS), water activity (*A_w_*), and moisture content (MC). Results of these baseline assessments served as a basis for comparison throughout storage.

Every 2 calendar months over a 24‐month period, samples from all treatment groups were assessed for PVs. When predetermined thresholds were reached for a sample set (PV > 2.0 meq active O_2_/kg oil), consumer panels evaluated these samples. In addition, 3 experienced sensory analysts evaluated the samples for indicators of quality degradation following ASTM (2011a) guidelines. This was done to ensure that any suspected deterioration in the samples was further evaluated, even if the PVs were below 2.0 meq active O_2_/kg oil. This procedure was followed to ensure that consumer sensory evaluations were conducted at appropriate times in the rejection period.

When threshold values were reached, the almonds were evaluated by a consumer screening panel (*n* = 35 to 40), and the panelists were asked about the acceptability of the sample as well as “If you had purchased this product would you eat it?” (Hough et al., 2003). If at least 75% of the panelists stated that they would eat the sample, the sample continued to be evaluated by a consumer panel at intervals of 2 calendar months until less than 75% of the panelists indicated they would consume the sample. If ≥25% of panelists stated that they would not consume the sample, these samples were then investigated with a larger confirmatory panel (*n* = 100 to 110) within 24 hours of the screening panel. If both screening and confirmatory panel rejection rate was ≥25%, the sample was deemed “failed,” and the data from the screening and confirmatory panel were pooled together for subsequent statistical analysis. At the points of their respective failures, samples were assessed by PV, FFA, CD, TBARS, *A_w_*, and MC. Following the chemical assessments at the time of failure, samples were no longer submitted for further sensory or chemical evaluations. At 24 months, all remaining stored samples were evaluated, even if the preliminary criteria for indication of quality deterioration had not been met.

### Almond characterization, handling, and packaging

The nuts investigated in this study were whole, raw, unsalted ‘Nonpareil’ almonds with brown skin. The almonds were a composite lot harvested from different orchards in California between September and October of 2014 and were graded “Supreme.” The almonds were pasteurized by propylene oxide fumigation prior to packaging. The nuts were shipped to the Dept. of Food Science & Technology, Athens, GA, U.S.A., in 50 lb cartons and the initial MC was determined to be 4.3%.

The raw almonds were repackaged at the Univ. of Georgia for storage in Uline S‐17960 (100 μm, clear polypropylene) bags or Uline S‐15138 corrugated cardboard boxes (Uline, Waukegan, IL, U.S.A.). The polypropylene material had a water vapor transmission rate of 8 g/m^2^d and oxygen transmission rate of 860 cm^3^/m^2^d, and the UC material provided no protection from atmospheric conditions. Each PPB was filled with 300 ± 5 g of raw almonds and then placed in a Model 600 vacuum packaging system (Henkelman B.V., The Netherlands). The filled bags were subject to vacuum for 30 s, and then flushed with food‐grade N_2_ prior to heat sealing, thereby forming a pillow pack for each sample. A total of 30 bags were filled per treatment. The initial oxygen level in the PPB was below 0.5%. For samples stored in cardboard boxes, 900 ± 5 g of raw almonds were placed in each box and then covered with a lid. A total of 12 boxes were used for each experimental treatment.

The almond samples receiving RH control were stored in Hotpack environmental chambers (Model 434304, SP Industries, Warminster, PA, U.S.A.). Some samples were also stored at 4 °C in a walk‐in cooler, but had no RH control. The temperature and RH of each chamber were monitored throughout the study.

### Sensory participants and demographic information

All participants for the sensory panels were over 18 years of age with no peanut or tree‐nut allergies. Ethnicity and sex were not controlled factors. Participants were recruited from faculty, staff, students, and visitors of the Univ. of Georgia. The criterion for inclusion was that the prospective panelist consumed nuts or nut products at least once per month. All participants were required to provide informed consent prior to participation. The demographics differed slightly for each panel, but across all panels, approximately 78% of the panelists were female with 73% between the ages of 18 and 27.

### Consumer sensory evaluation

The sensory evaluation protocol followed ASTM methodology (ASTM, 2011b). For all evaluations, sample cups were coded with random 3‐digit codes and served with 3 almonds per sample cup. The panelists were seated in individual sensory booths equipped with white lighting. The samples were presented one at a time in a counterbalanced order of presentation. Water and baby carrots were provided as palate cleansers.

For all consumer evaluations, the panelists evaluated the almond samples for acceptability of odor, texture, and flavor as well as overall acceptability by rating the samples on a 9‐point hedonic scale. The scale ranged from “extremely dislike” (1) to “extremely like” (9). The panelists were also asked to indicate the favorable and unfavorable traits of each sample (Rousset & Martin, 2001), by responding to the question “*Please indicate WHAT in particular you liked or disliked about this almond sample (use WORDS not SENTENCES)*.” Finally, the panelists were asked to respond to “*If you had purchased this product, would you eat it? (yes or no)*” and a negative response to this question was defined, for our study, as equivalent to “rejection” of the sample (Hough et al., 2003).

### Chemical analyses

All chemical assessments were performed in triplicate. PVs were determined for all samples at intervals of 2 calendar months throughout the study according to AOAC Method 965.33 (AOAC, 2012). FFA, CD, TBARS, *A_w_*, and MC were determined for the samples at baseline and for samples following their “failure” (according to consumer assessment). Samples that did not fail during the study were assessed at the conclusion of the 24‐month period.

FFAs were determined according to AOCS Method Ca 5a‐4020 (AOCS, 2009). CDs were determined according to IUPAC Official Method 2.505 (IUPAC, 1992). TBARS were determined according to AOCS Official Method Cd 19–90 (AOCS, 2009). *A_w_* was determined by loading 2 g (±0.1 g) of the ground almond meal into a calibrated Aqua Lab CX‐2 water activity meter (Pullman, WA, U.S.A.). MC (percentage value of moisture mass within total sample mass) was determined for ground almonds by assessment of mass loss following heating in a forced‐air convection oven at 105 °C until constant weight was achieved (AOAC, 2012).

### Statistical analysis

All statistical analyses were performed with SAS 9.4 Statistical Analysis Software (SAS Inst. Inc., Cary, NC, U.S.A.). Normal distribution was verified through univariate analysis. Descriptive statistics were determined for assessments and *t*‐tests were conducted to test significant changes over time when compared to baseline. Analysis of variance (ANOVA) with Student–Newman–Keuls (SNK) was conducted to detect significant differences between samples at the point of rejection.

Multiple regression analyses were conducted to model acceptability of individual sensory attribute (odor, texture, and flavor) as predictors of overall acceptability. Multiple regression models were also developed for the prediction of the time of failure according to storage parameters and these modeling procedures excluded samples that did not fail during the duration of the study. Additional models were made for the prediction of each of the 4 variables representing consumer acceptability (odor, flavor, texture, and overall) according to the assessed chemical measures, utilizing the data assessed at baseline and at sample failure (or study conclusion) for each of the samples.

The multiple regression models used in this study were developed using the “Best Subsets” procedure. In essence, 8 models were evaluated using combinations of the predictors (temperature, RH, or packaging). Of these, the best fit from each of the 1‐term, 2‐term, or 3‐term models was selected based on the *R* ^2^ value. From these models, the one with the greatest adjusted *R* ^2^ value was selected. The adjusted *R* ^2^ is related to the mean square error, and thus penalizes the model for adding additional terms. For all statistical assessments, the level of statistical significance was defined at *α* = 0.05.

## Results and Discussion

### Assessment of raw almonds at baseline

The baseline assessments (day 0) of PVs (<0.01 meq active O_2_/kg oil), FFA (0.28 acid value), CD (1.43), TBARS (0.030), *A_w_* (0.29), and MC (3.1%) of the raw almonds suggested the almonds met industry‐standard criteria for freshness of raw almonds (Almond Board of California, 2015). Specifically, the PVs, FFAs, and MCs were all below the recommended maximum values for verification of freshness (5 meq active O_2_/kg oil, 3.0 acid value, and 3.5% to 6% MC, respectively). The FFA and PV levels were also below those reported for raw almonds prior to lipid deterioration (Lin et al., 2012; Mexis & Kontominas, 2010). In addition, the *A_w_* of the almonds fell within the 0.25 to 0.35 range at which lipid oxidation is typically lowest (Huang, 2014).

The baseline consumer sensory assessments of odor, texture, flavor, and overall acceptability of the raw almonds are shown in Table 2. Mean texture, flavor, and overall acceptability were above 7 on the 9‐point hedonic scale, while mean acceptability of odor was 5.7. It is important to note that consumers were instructed to evaluate the odor of the sample prior to masticating the almonds; thus, it is probable that fewer volatile compounds were detected than would have been during mastication. In addition, as the almonds were raw, it may be that some consumers were expecting odors more characteristic of roasted almonds. Mean texture, flavor, and overall acceptability at baseline can reasonably be interpreted as indicating the product was free of substantial defects or detriments.

**Table 2:** Sensory results^a^ for samples at baseline and point of failure/end of study

			Odor^b^	Texture^b^	Flavor^b^	Overall acceptability^b^
		Point of failure	Means ± Standard deviations^c^			
Baseline			5.7 ± 1.5 a	7.2 ± 1.5 a	7.3 ± 1.4 a	7.2 ± 1.4 a
Polypropylene bags	4 °C		5.3 ± 1.4 a	6.2 ± 1.9 bc	6.6 ± 1.8 b	6.5 ± 1.7 cd
	15 °C/50% RH		5.7 ± 1.5 a	6.6 ± 1.8 b	6.8 ± 1.6 b	6.8 ± 1.5 ab
	15 °C/65% RH	24 mo	5.4 ± 1.7 a	5.4 ± 2.2 def	5.3 ± 2.1 cd	5.4 ± 2.1 de
	25 °C/50% RH		5.1 ± 1.8 a	6.0 ± 1.9 bcd	5.8 ± 2.0 c	5.9 ± 2.0 cd
	25 °C/65% RH	16 mo	5.3 ± 1.8 a	5.8 ± 2.0 cde	5.8 ± 2.1 cd	5.7 ± 2.0 d
	35 °C/50% RH	12 mo	5.2 ± 1.6 a	6.4 ± 1.8 bc	5.7 ± 2.1 cd	5.9 ± 1.9 cd
	35 °C/65% RH	6 mo	5.5 ± 1.7 a	5.9 ± 2.1 bcd	5.7 ± 2.3 cd	5.8 ± 2.1 d
Unlined cartons	4 °C	6 mo	4.6 ± 1.7 b	4.3 ± 2.3 g	5.6 ± 2.1 cd	5.0 ± 2.3 e
	15 °C/50% RH	16 mo	5.4 ± 1.8 a	5.1 ± 2.3 f	5.6 ± 2.1 cd	5.5 ± 2.0 de
	25 °C/50% RH	16 mo	5.6 ± 1.6 a	5.8 ± 2.0 cde	5.6 ± 1.8 cd	5.8 ± 1.7 d
	25 °C/65% RH	12 mo	5.3 ± 1.5 a	5.2 ± 2.1 ef	5.0 ± 2.1 d	5.2 ± 1.9 de
	35 °C/50% RH	6 mo	5.6 ± 1.6 a	5.8 ± 2.0 cde	5.6 ± 2.1 cd	5.7 ± 2.0 d
	35 °C/65% RH	2 mo	5.3 ± 1.4 a	5.5 ± 2.2 def	5.2 ± 2.1 cd	5.4 ± 2.1 de

a Data from screening and confirmatory panels combined (*n* > 115).

b Hedonic scale where 1 is “extremely dislike” and 9 is “extremely like.”

c Means ± standard deviations followed by different letters within a column differ significantly

(*P* < 0.05) according to ANOVA and SNK means.

At baseline, roughly 6% of the consumer panelists rejected the raw almond sample. Considering the freshness of the almonds, one might expect that no panelists would reject the samples. However, previous studies have found that very fresh almonds can result in a perception of unacceptable quality by some consumers (Hough et al., 2003). It has been postulated that some consumers may be more accustomed to eating older/aged almonds (such as those found in the bulk bins at grocery stores), therefore making the absence of aged and oxidized flavor notes an unexpected, and perhaps undesirable, characteristic.

At baseline, roughly ½ of the panelists described the almonds as “crunchy,” using this term to describe something they liked about the sample. Most panelists also stated that they liked the overall texture and flavor/taste of the sample, with the most commonly used words to describe the flavor being “nutty” and “sweet.” The odor was not listed as a quality that was liked by many panelists. The most common specific complaint regarding odor was that the sample had a “weak” or “mild” odor. The most common complaint about flavor for the sample at baseline was that the sample was “bland” or “mild,” while the most common textural complaint was that it was “hard” and “dry.” These quality concerns for flavor and odor are consistent with the hypothesis that panelists may be responding to the absence of roasted or positive oxidized flavor notes.

### Consumer sensory evaluation of almonds stored in polypropylene bags

Table 2 summarizes the consumer sensory results of all samples at their final assessment and includes statistical comparisons with one another and with baseline. Of the 7 samples stored in PPB, consumer panelists rejected 4 of the samples prior to the conclusion of the study. All samples stored at 65% RH were rejected, with those stored at 35 °C rejected at 6 months, those at 25 °C at 16 months, and those at 15 °C at 24 months. In addition, the sample stored at 35 °C and 50% RH was rejected at 12 months. The 2 samples stored at 35 °C were the first to be rejected from the study, suggesting a substantial influence of temperature on consumer acceptability and rejection. Although all samples were “triggered” for consumer evaluation within 12 months, 3 of the samples did not fail within the timeframe of the study (that is, those at 4 °C, 15 °C/50% RH, and 25 °C/50% RH).

The sample most similar to baseline at the end of the study was that stored in PPB at 15 °C/50% RH. The scores for odor and overall acceptability for this sample were not significantly different from baseline when the study concluded at 24 months, and although the flavor was significantly lower when compared to baseline, it had the highest score of all samples. This indicates that the quality of raw almonds is best maintained when stored in PPB, which provide a barrier to both oxygen and water vapor transmission, and when stored at low temperature and humidity. Although at lower temperature, almonds stored at 4 °C had lower acceptability than those at 15 °C. This is likely due to the uncontrolled RH in the walk‐in cooler, which could reach up to 90% humidity at times during the study.

In general, acceptability scores for odor were not significantly different from baseline values throughout the 24‐month storage period. In contrast, significant differences in texture, flavor, and overall acceptability (against their respective baseline values) were observed for all rejected samples at their points of failure.

### Consumer sensory evaluation for almonds stored in unlined cartons

Consumer panelists rejected all samples stored in UC at some point during the 24‐month storage period (Table 2). In order, those at 35 °C/65% RH were rejected at 2 months, those at 35 °C/50% RH at 6 months, those at 4 °C at 6 months, those at 25 °C/65% RH at 12 months, and those at 25 °C/50% RH or 15 °C/50% RH at 16 months. Mean panelist responses for the acceptability attributes for these samples were consistently lower than the scores for their counterparts stored in PPB.

Samples stored in UC at 4 °C exhibited the greatest quality deterioration of all samples. These had significantly lower scores for odor, texture, and overall acceptability than did all other samples. However, the flavor acceptability score for this sample differed significantly only from 2 samples stored in PPB (4 °C and 15 °C/50% RH). No differences between this sample and the remaining samples stored in UC were found.

When comparing acceptability scores for all attributes against those at baseline, significant differences were seen for more than half of the sensory results. This highlights that almonds stored in UC demonstrated a greater reduction in sensory quality than those stored in PPB. Analysis of the strong and weak points stated by panelists for the samples at the point of failure showed that samples stored in UC were more frequently described as having a “cardboard” flavor or odor, especially among individuals who rejected the samples. Although “cardboard” is a term that is associated with lipid oxidation, it is feasible that these almonds developed detrimental cardboard flavor notes *via* direct interaction with the cardboard boxes.

### Effects of storage parameters on consumer assessments

Table 3 shows the multiple regression model for the prediction of time (in months) until sample failure, according to storage parameters. The model has a fairly strong predictive strength (adjusted *R* ^2^ = 78.9%) and depicts quantitatively the effects of the assessed storage parameters on raw almond stability. The model predicts that storage of raw almonds in PPB (rather than UC) would prolong consumer acceptability by 7.21 months. This finding generally agrees with an examination of the results, as for each storage condition, every sample packaged in UC failed prior to those in PPB stored under equivalent conditions. This relative protective effect of PPB is expected, as UC provide minimal protection against moisture or oxygen transfer and may also lead to more rapid quality deterioration due to the migration of flavor‐imparting compounds from the packaging to the product (Guinard & Mazzucchelli, 1996).

**Table 3:** Multiple linear regression model for time of failure according to storage parameters.^a^

	Linear regression coefficients				
Value	Intercept	PPB^b^	T(°C)	RH%	*R* ^2^(adj)
Time of failure (months)	49.1	7.21	−0.589	−0.418	78.9%

a Modeling excluded samples that did not fail during the 24‐month assessment period.

b A binary term for which [0 = almonds stored in UC] and [1 = almonds stored in PPB].

The model in Table 3 also shows a negative effect of T and RH on sample stability. García‐Pascual, Mateos, and Salazar (2003) reported that there was a significant increase in shelf life of roasted almonds by decreasing the storage temperature from 36 to 8 °C. The authors suggested that there is a protective effect of temperature reduction when samples are exposed to high humidity. Specifically, our model predicts the time until failure is reduced by 0.589 months for each additional °C of storage T (within the assessed range). The model also predicts time until failure is decreased by 0.418 months with each 1% increase in RH. The effects suggested by the model are compatible with an examination of the results, as storage at 65% RH resulted in reduced/shorter shelf life than storage at 50% RH in both packaging materials. At 25 and 35 °C, this 10% reduction in RH was associated with measured extensions in the shelf life of 4 and 6 months, respectively.

Table 4 shows statistical comparisons between samples stored in PPB versus those in UC (but otherwise identical conditions) for the acceptability scores at the time of failure/end of study. At the lower storage temperatures (4 and 15 °C), acceptability scores of all attributes differed significantly between the samples stored in PPB versus UC, whereas for those stored at 35 °C, only texture differed significantly. Specifically, texture acceptability was significantly higher for almonds stored in PPB at 35 °C (6.3 ± 1.9 for 50% RH and 6.1 ± 1.9 for 65% RH) than those stored in UC at 35 °C (5.7 ± 1.9 for 50% RH and 5.5 ± 2.2 for 65% RH). This suggests that the chemically mediated attributes of flavor and odor are more susceptible to changes in temperature. Thus, limiting moisture and oxygen transfer through polypropylene packaging may not effectively counter the increase in chemical reactions due to temperature. However, texture attributes are more susceptible to moisture absorption, as water softens the structure and decreases the fracturability associated with firm, crunchy nuts.

**Table 4:** Comparison of polypropylene bag versus unlined carton for each storage condition for acceptability of odor, texture, flavor, and overall at point of failure/end of study

	Odor	Texture	Flavor	Overall acceptability
4 °C	*	*	*	*
15 °C/50% RH	*	*	*	*
25 °C/50% RH	*			
25 °C/65% RH				
35 °C/50% RH		*		
35 °C/65% RH		*		

^*^ndicates significant difference (*P* < 0.05) between samples stored in bags versus cartons held under the same storage conditions.

### Specific hedonic measures as predictors of overall consumer acceptability

Multiple regression analysis was used to identify the contribution of each attribute as a predictor of overall acceptability for each of the sensory panels (Table 5). The table presents results for each time and set of conditions in which a sensory panel was conducted. At baseline, all 3 sensory attributes were found to be predictors of overall acceptability (*R* ^2^ = 0.80), with contributions of flavor, texture, and odor to overall acceptability being 65.7%, 13.1%, and 1.4%, respectively.

**Table 5:** Contribution of attributes to overall acceptability by multiple regression.^a^

Storage condition		Testing point		*n*	Rejection rate %^b^	*R* ^2^	Odor^c^	Texture^c^	Flavor^c^
Baseline				117	6.0	0.80	0.0138	0.1313	0.6574
Polypropylene bags	4 °C	12 mo	screen	36	11.1	0.90	X	0.5764	0.3213
		14 mo	screen	37	11.1	0.89	X	0.1045	0.7816
		16 mo	screen	35	5.7	0.93	X	0.1000	0.8289
		18 mo	screen	34	8.8	0.87	X	0.0658	0.8041
		20 mo	screen	37	13.5	0.90	X	0.1048	0.7904
		22 mo	screen	38	8.1	0.65	X	0.0604	0.5881
		24 mo	screen	38	2.7	0.85	X	0.1309	0.7165
			confirm	101	12.9	0.89	X	0.0532	0.8366
	15 °C/50% RH	12 mo	screen	36	11.1	0.78	X	0.1713	0.6037
		14 mo	screen	37	13.9	0.84	0.0436	0.0872	0.7137
		16 mo	screen	35	8.6	0.78	X	0.1044	0.6802
		18 mo	screen	36	5.7	0.79	0.0505	0.0842	0.6576
		20 mo	screen	37	8.3	0.92	0.0123	0.6788	0.2249
		22 mo	screen	38	13.9	0.89	X	0.1339	0.7565
		24 mo	screen	36	16.7	0.89	X	0.1727	0.7217
			confirm	94	10.6	0.79	X	0.1508	0.6398
	15 °C/65% RH	12 mo	screen	36	8.3	0.95	X	0.1069	0.8451
		14 mo	screen	35	11.4	0.92	X	0.0299	0.8927
		16 mo	screen	36	30.6	0.87	X	0.1642	0.7067
			confirm	100	22.0	0.86	0.0160	0.0959	0.7530
		18 mo	screen	35	5.7	0.74	X	0.1406	0.5956
		20 mo	screen	37	21.6	0.86	X	0.0584	0.8012
		22 mo	screen	34	17.7	0.88	0.0197	0.0243	0.8330
		24 mo	screen	38	60.5	0.84	X	0.1087	0.7337
			confirm	97	29.9	0.90	0.0219	0.1199	0.7552
	25 °C/50% RH	12 mo	screen	37	16.2	0.84	X	X	0.8396
		14 mo	screen	36	19.4	0.92	0.0117	0.0458	0.8622
		16 mo	screen	35	17.1	0.82	X	0.0968	0.7233
		18 mo	screen	36	19.4	0.90	X	0.0697	0.8262
		20 mo	screen	36	11.1	0.88	X	0.1094	0.7673
		22 mo	screen	37	10.8	0.94	0.0091	0.0449	0.8839
		24 mo	screen	35	31.4	0.90	0.0169	0.1111	0.7672
			confirm	100	23.0	0.91	0.0055	0.0522	0.8505
	25 °C/65% RH	12 mo	screen	35	17.1	0.83	X	0.0657	0.7681
		14 mo	screen	36	19.4	0.83	X	0.1530	0.6787
		16 mo	screen	35	28.6	0.93	X	0.0990	0.8267
			confirm	101	28.7	0.91	0.0092	0.0541	0.8422
Polypropylene bags	35 °C/50% RH	6 mo	screen	35	11.4	0.74	X	0.2040	0.5338
		8 mo	screen	34	14.7	0.89	X	0.1172	0.7722
		10 mo	screen	37	21.6	0.90	X	0.0355	0.8634
		12 mo	screen	35	31.4	0.89	X	0.1097	0.7754
			confirm	98	28.6	0.87	X	0.0505	0.8166
	35 °C/65% RH	2 mo	screen	38	10.5	0.79	X	0.0303	0.7611
		4 mo	screen	34	14.7	0.83	X	0.1252	0.7004
		6 mo	screen	35	34.3	0.81	X	0.1249	0.6850
			confirm	92	27.2	0.91	0.0063	0.0581	0.8466
Unlined cartons	4 °C	2 mo	screen	39	20.5	0.84	X	0.7601	0.0768
		4 mo	screen	37	13.5	0.74	X	0.1949	0.5406
		6 mo	screen	36	47.2	0.93	X	0.8583	0.0735
			confirm	91	35.2	0.68	0.0159	0.0853	0.7636
	15 °C/50% RH	10 mo	screen	37	16.2	0.82	X	0.0837	0.7395
		12 mo	screen	37	21.6	0.82	X	0.1200	0.6961
		14 mo	screen	35	17.1	0.83	X	0.1761	0.6516
		16 mo	screen	36	30.6	0.91	X	0.1300	0.7848
			confirm	100	40.6	0.92	X	0.1147	0.8048
	25 °C/50% RH	12 mo	screen	35	22.9	0.91	X	0.1865	0.7228
		14 mo	screen	35	8.6	0.90	X	0.1457	0.7523
		16 mo	screen	35	28.6	0.81	X	0.1035	0.7035
			confirm	97	28.9	0.88	0.0182	0.1544	0.7068
	25 °C/65% RH	12 mo	screen	36	61.1	0.83	0.0281	0.1012	0.7023
			confirm	96	36.5	0.86	X	0.0780	0.7836
	35 °C/50% RH	6 mo	screen	36	41.7	0.90	X	0.0219	0.8749
			confirm	91	29.7	0.87	X	0.0567	0.8106
	35 °C/65% RH	2 mo	screen	40	30.0	0.90	X	0.0707	0.8288
			confirm	110	32.7	0.89	X	0.1062	0.7883

a Significant contribution (*P* < 0.05) determined using multiple regression; values listed are the partial *R* ^2^ of each attribute.

b Negative response to “If you had purchased this product would you eat it?” (yes or no).

c X in column indicates attribute was not a significant predictor and was therefore excluded from the model.

For 65 of the 69 models, the flavor was the largest predictor of overall acceptability, ranging from 53% to 89% (71% to 85% for samples at the point of failure). Texture was the largest predictor for the 4 remaining instances (PPB at 4 °C at 12 months, UC at 4 °C at 2 and 6 months, and PPB at 15 °C/50% RH at 20 months); it is worth noting that for each of these, the models were based upon screening panels, and thus had smaller sample sizes than the confirmatory panels. The odor was the weakest predictor of overall acceptability for every model produced, and it was omitted from the model in more than half of the cases due to lack of significance. In no cases did the odor variable contribute more than 5.1% predictive strength to the model.

The importance of individual attributes to overall acceptability was also analyzed using multiple regression models of pooled consumer data (Table 6). Among these are models for 3 divisions of the data—one using consumer data for all panelists and samples, one using consumer data for panelists that rejected the sample, and one using the data from panelists that accepted the sample. Table 6 lists the parameter estimates for the intercepts as well as for each of the individual sensory attributes and indicates the partial *R* ^2^ for each sensory attribute and the cumulative *R* ^2^. Odor, texture, and flavor were all significant factors in predicting overall acceptability. The model created for all sensory data explained the most variability (87.3%), while that created based on rejected samples explained the least (75.1%). In all 3 models, flavor was the largest determinant of overall acceptability and odor was the smallest. For example, considering all data partial *R* ^2^ values were 0.774, 0.093, and 0.0055 for flavor, texture, and odor, respectively. For panelists who rejected the samples, texture and odor were more important, although flavor was still a dominant factor. Thus, the partial *R* ^2^ values were 0.564, 0.168, and 0.0183 for flavor, texture, and odor, respectively.

**Table 6:** Contribution of attributes to overall acceptability by multiple regression models.^a^

		Intercept	Odor^d^	Texture^d^	Flavor^d^	*R* ^2^
Overall acceptability^d^ among all panelists (*n* = 3326)	Parameter estimate	0.030	0.097	0.326	0.591	
	Partial *R* ^2^		0.0055	0.0930	0.774	0.873
Overall acceptability^d^ among panelists accepting the sample (*n* = 2586)^b^	Parameter estimate	0.553	0.083	0.311	0.549	
	Partial *R* ^2^		0.0062	0.119	0.698	0.825
Overall acceptability^d^ among panelists rejecting the sample (*n* = 740)^c^	Parameter estimate	−0.076	0.132	0.308	0.538	
	Partial *R* ^2^		0.0183	0.168	0.564	0.751

a Significant contribution (*P* < 0.05) determined using multiple regression.

b Panelists who responded “yes” to “If you had purchased this product would you eat it?”

c Panelists who responded “no” to “If you had purchased this product would you eat it?”

d Based on hedonic scale where 1 is “extremely dislike” and 9 is “extremely like.”

The observed differences in the models for panelists who accepted versus those who rejected the samples suggest consumers may use different criteria when evaluating “intent to consume.” Specifically, odor and texture appear to be more important determinants for panelists who reject samples than panelists who do not, indicating that undesirable odors and textures played a larger role than desirable ones in predicting consumer rejection. On the other hand, flavor played a greater role in predicting consumer acceptability.

### Chemical measures as predictors of consumer assessment

The results of the chemical assessments for the samples at the time of failure (or at 24 months in the absence of failure) are presented in Table 7. As expected, chemical measures of deterioration increased for the samples during storage. An examination of the average values within the 2 packaging types shows the samples in UC, in sum, to have significantly higher *A_w_* at time of failure (or conclusion of study; *P* = 0.021) than those in PPB. This is reasonable, considering the lack of vapor protection provided by UC. Although the average value of MC is also higher for UC than for PPB, neither for this measure nor any of the other assessed chemical measures were the averages within package type significantly different from one another.

**Table 7:** Chemical assessments for samples at point of failure/end of study (*n* = 3)

		Point of failure	PV (meq active O_2_/kg oil)	FFA (acid value)	CD	TBARS	*A_w_*	MC
Polypropylene bags	4 °C		2.39	0.302	2.4	0.0084	0.43	4.7%
	15 °C/50% RH		2.27	0.404	6.1	0.0099	0.46	5.0%
	15 °C/65% RH	24 mo	1.87	0.633	4.4	0.0095	0.49	5.2%
	25 °C/50% RH		1.95	0.690	8.6	0.0076	0.41	4.1%
	25 °C/65% RH	16 mo	4.15	0.538	4.6	0.0097	0.53	3.7%
	35 °C/50% RH	12 mo	4.26	0.681	8.1	0.026	0.50	3.9%
	35 °C/65% RH	6 mo	1.40	0.591	4.9	0.031	0.56	4.3%
	Average		2.61	0.548	5.6	0.015	0.48	4.4%
Unlined cartons	4 °C	6 mo	4.05	0.331	3.2	0.054	0.77	6.8%
	15 °C/50% RH	16 mo	4.30	0.334	2.1	0.0083	0.54	4.5%
	25 °C/50% RH	16 mo	3.64	0.440	5.8	0.0095	0.48	3.9%
	25 °C/65% RH	12 mo	4.05	0.708	2.5	0.030	0.62	5.5%
	35 °C/50% RH	6 mo	2.10	0.597	4.8	0.055	0.56	4.4%
	35 °C/65% RH	2 mo	2.68	0.990	3.6	0.057	0.62	5.8%
	Average		3.47	0.567	3.7	0.036	0.60	5.2%

Table 8 shows the predictive models for consumer outputs of odor, flavor, texture, and overall acceptability according to assessed chemical factors. The correlation coefficients suggest decent predictive strengths for these models. Specifically, the model for consumer assessment of odor had the greatest predictive strength (adjusted *R* ^2^ = 71.1%) and the model for flavor assessment had the lowest (adjusted *R* ^2^ = 53.5%). The variable coefficients of the models are all within expectation. It is notable that PV and FFA both associate negatively with the assessment of flavor, odor, and overall acceptability, while the CD and TBARS variables failed to improve these models. This suggests that the chemical assessments of PV and FFA in raw almonds may be of particular utility for the evaluation of raw almond quality throughout storage. The models show that increases in PV were associated with a greater reduction in expected odor assessment (−0.696 reduction for each meq active O_2_/kg oil) than for expected assessments of flavor or overall acceptability (−0.174 and −0.143 reductions for each meq active O_2_/kg oil, respectively). Conversely, the modeled effects of FFA were of lower magnitude for odor than for flavor or overall acceptability.

**Table 8:** Summary of multiple linear regression models of sensory^a^ values versus chemical assessments

		Linear regression coefficients						
Value	Intercept	PV^b^	FFA^c^	CD	TBARS	*A_w_*	MC	*R* ^2^(adj)
Odor	5.83	−0.696	−0.413	d	d	d	d	71.1%
Flavor	8.70	−0.174	−1.56	d	d	−2.93	d	53.5%
Texture	9.09	d	d	d	d	−4.40	−0.22	69.3%
Overall	8.75	−0.143	−0.756	d	d	−3.98	d	62.0%

a Hedonic scale where 1 is “extremely dislike” and 9 is “extremely like”; data from screening and confirmatory panels combined (*n* > 115).

b meq Active O_2_/kg oil.

c Acid value.

d Omitted from model due to failure to improve model according to adjusted *R* ^2^.

The most predictive model for assessed texture quality contained exclusively the factors of *A_w_* and MC. For both variables, the coefficients show negative associations with predicted texture quality, indicating deleterious effects of increases in total and available water in raw almonds. This provides further evidence of the possible usefulness of controlling storage humidity, and for storing raw almonds in packaging that mitigates water vapor transmission.

In addition to the noted observed effect of *A_w_* on textural quality, *A_w_* was also determined to be a useful variable in the prediction of overall acceptability and flavor acceptability. The effect on overall acceptability is unsurprising. The effects on flavor may be secondary. That is, it is known that the rates of lipid oxidation are dependent upon the *A_w_*/MC of nut products. As the *A_w_* rises above 0.2 to 0.3, lipid oxidation can occur more expediently as reactive molecules become more mobile. In addition, there may also be multimodal sensory integration by panelists, with the panelists’ assessments of flavor influenced directly by textural qualities (Forde & Delahunty, 2004).

## Conclusions

Models revealed that flavor was the greatest contributor to determining consumer acceptability, followed by texture. Odor provided only a comparatively small contribution. It was also found that PVs and FFAs were of greater importance in predicting raw almond consumer quality than measures of CD or TBARS. Measures of *A_w_* and MC were the best predictors of consumer assessments of textural quality

Increases in storage T and RH were deleterious to raw almond quality during storage, decreasing substantially the time until a sample's failure by consumer assessment. The use of PPB packaging (rather than UC) was found to substantially mitigate quality losses throughout storage, with PPB packaging increasing the time of storage until failure by approximately 7 months. Samples stored in UC always failed before their counterparts stored in PPB under identical environmental conditions. Moreover, acceptability scores of almonds stored in UC were frequently lower than their counterparts stored in PPB. The importance of these factors should be seriously considered when determining packaging strategies, and further studies should be conducted on methods to improve the preservation of almond quality. Packaging materials with oxygen and moisture barriers superior to those of PPB may be studied to determine if the shelf life of raw almonds could be further extended.

## Conflict of Interest

The authors declare that they have no conflict of interest.

## Author Contributions

Emily A. Pleasance performed the sensory evaluation, analyzed results, and wrote much of the text.

William L, Kerr designed the storage conditions and coordinated physical measurements of the raw almonds.

Ronald B. Pegg supervised all chemical analyses of samples.

Ruthann B. Swanson coordinated the sensory evaluation and data analysis.

Anna N. Cheely performed sensory evaluation and analyzed results.

Guangwei Huang determined study objectives and study design in accordance with the needs of the almond industry.

Daniel R. Parrish performed the chemical analyses.

Adrian L. Kerrihard contributed data analysis and interpretation, writing, and editing.
